# Association of periodontal disease with depression and adverse birth outcomes: Results from the Perinatal database; Finger Lakes region, New York State

**DOI:** 10.1371/journal.pone.0215440

**Published:** 2019-04-18

**Authors:** Dorota T. Kopycka-Kedzierawski, Dongmei Li, Jin Xiao, Ronald J. Billings, Timothy D. Dye

**Affiliations:** 1 Department of Dentistry, Eastman Institute for Oral Health, University of Rochester, Rochester, NY, United States of America; 2 Department of Clinical and Translational Research, University of Rochester, Rochester, NY, United States of America; 3 Department of Obstetrics and Gynecology, University of Rochester, Rochester, NY, United States of America; Univesity of Iowa, UNITED STATES

## Abstract

Preterm and low birth weight infants are at greater risk for mortality and a variety of health and developmental problems. Data from the Finger Lakes Perinatal Data System database on 316,956 deliveries occurring between 2004–2014 and pregnancy outcomes were analyzed to assess the association of periodontal (gum) disease with depression, other maternal factors and adverse birth outcomes. Adjusted effects of periodontal disease and depression on adverse birth outcomes were estimated using multiple logistic regression models and path analysis. Having preterm delivery was associated significantly with depression (OR = 1.177; 95% CI: [1.146, 1.208]), having adequate health care (OR = 1.638; 95% CI: [1.589, 1.689]), smoking during pregnancy (OR = 1.259; 95% CI: [1.220, 1.300]), and being less educated (OR = 1.214; 95% CI: [1.174, 1.256]). Having low birth weight was significantly associated with depression (OR = 1.206; 95% CI: [1.170, 1.208]), smoking during pregnancy (OR = 1.855; 95% CI: [1.793, 1.919]), and being less educated (OR = 1.322; 95% CI: [1.275, 1.370]). Periodontal disease was significantly associated with alcohol use during pregnancy (OR = 1.314; 95% CI: [1.227, 1.407]) and white race (OR = 1.192; 95% CI: [1.167, 1.217]). Depression was significantly associated with periodontal disease (OR = 1.762; 95% CI: [1.727, 1.797]) and alcohol use during pregnancy (OR = 1.470; 95% CI: [1.377, 1.570]). We concluded that a positive association existed between depression during pregnancy and adverse birth outcomes, and that depression served as a mediator in the association of periodontal disease with adverse birth outcomes.

## Introduction

Maintaining good oral health during pregnancy is an important aspect of maintaining overall good health during pregnancy and throughout a woman’s lifespan. According to the Advisory Committee for Oral Health Care During Pregnancy, in years 2007–2009, 35% of U.S. women did not attend a dental visit within the past year and 56% of women did not visit a dentist during pregnancy [1]. Numerous studies have shown positive associations of periodontal disease during pregnancy with preterm delivery, low birth weight, low weight for gestational age and increased risk for preeclampsia [2–6]. However, other studies failed to find an association [7, 8]. In a prospective cohort study of pregnant women conducted in North Carolina, periodontal health status and changes in oral health that occurred during pregnancy were assessed. Periodontal disease during pregnancy was reported to be most prevalent among women who were African American, cigarette smokers, and users of public assistance programs. Women with moderate/severe periodontal disease at enrollment were more likely to experience incident disease when compared to those with no disease at enrollment [9].

According to the literature, maternal depression during pregnancy is also associated with adverse perinatal and infant outcomes. A systematic review and meta-analysis of thirty studies suggested that premature delivery was significantly associated with maternal depression [10]. A large multi-ethnic study conducted in Holland in 2003 on 7,740 pregnant women reported that babies of pregnant women with high levels of anxiety and depressive symptoms were at the highest risk for adverse birth outcomes, including a lower birth weight and an increased risk for pre-term birth [11]. A population based cohort study conducted among women of the Kaiser Permanente Medical Care Program to examine the impact of prenatal depression showed that pregnant women with depressive symptoms had almost twice the risk of preterm delivery when compared with women without depressive symptoms and the risk of preterm delivery increased with increasing severity of depression [12]. Data from the National Health and Nutrition Examination Survey (NHANES 2005–2008) were used to examine the relationship between poor dental health and depression among US adults controlling for markers of inflammation, C-reactive protein (CRP) and adiposity. Based on logistic regression analysis, a positive association was found between poor dental health and depression that was independent of CRP and Body Mass Index (BMI) [13].

Poor oral health, especially gingivitis and periodontitis, is recognized as a source of systemic inflammation. Currently, there is emerging evidence that depression is also an inflammatory disorder, indicated by increased levels of CRP and other pro-inflammatory factors; persons diagnosed with depression have been found to have higher levels of proinflammatory cytokines, acute phase proteins, chemokines and cellular adhesion molecules. Additionally, proinflammatory cytokines have been found to interact with many of the pathophysiological domains that characterize depression, including neurotransmitter metabolism, neuroendocrine function, synaptic plasticity and behavior [14]. There are several established risk factors for preterm birth including parity, smoking, alcohol, early and advanced maternal age and socioeconomic variables [15] .

Considering similar mechanisms related to systemic inflammation that are associated with depression and periodontal disease, we hypothesize that there is a positive association between periodontal disease and adverse birth outcomes and there is a positive association between depression and adverse birth outcomes. Further, we examine the positive association of periodontal disease with adverse birth outcomes mainly through the mediating effect of depression. A mediator is a variable that is in a causal sequence between two variables [16]. Mediating variables are prominent and often used in psychological theory and research.

In this study, we therefore sought to examine the association between periodontal disease and depression and between adverse birth outcomes among women who gave birth between 2004–2014 in thirty counties surrounding Rochester and Syracuse, New York.

## Methods

The Perinatal Data System (PDS) initiative began in New York State (NYS) in the early 1990s as a perinatal quality improvement initiative funded by the New York State Department of Health. The Upstate New York Regional Perinatal Centers in Albany, Syracuse, Rochester, and Buffalo were among the first demonstration centers for the regionalization of perinatal services in the United States; all evolved into NYS-supported Regional Perinatal Centers with both quality of care and regional perinatal service responsibilities. The PDS effort was envisioned to support a data infrastructure to serve the quality improvement and evaluative needs of the Perinatal Centers, resulting in the PDS demonstration projects starting in 1993. The PDS database includes information on demographics of both infants and parents, access to care, pre-pregnancy risks, antepartum risky behaviors, antepartum infections, antepartum risks, maternal and birth outcomes. The PDS data used for the current analysis was collected between 2004 and 2014. The PDS database contains data on more than 345,000 deliveries that occurred between 2004 and 2014 in thirty counties surrounding Rochester and Syracuse and includes data on pregnancy outcomes; data on 316,956 deliveries formed the basis for the analyses. The University of Rochester Research Subject Review Board (RSRB) approved the study prior to its initiation. The data used in the study were fully anonymized before retrieval.

## Description of measures used in the analyses

The database includes a dichotomous survey question asked of all postpartum mothers that states: “Did you have any problems with your gums at any time during pregnancy, for example, swollen or bleeding gums?” The depression information was collected using the following question in the PDS survey questionnaire: “During your pregnancy, would you say that you were (select one): Not depressed at all, A little depressed, Moderately depressed, Very depressed, Very depressed and had to get help”. The depression question was adopted from the CDC’s PRAMS (Pregnancy Risk Assessment and Monitoring System) [16–19]. The CDC initiated PRASMS in 1987 to provide state-specific, population-based surveillance of selected maternal behaviors that occur before, during and after pregnancy. The variables related to gestational age were defined as follows: SGA-small for gestational age (a binary variable), was defined as less than 10 percentile of the birth weight among infants with same gender and gestational age. LGA- large for gestational age (a binary variable), was defined as greater than 90 percentile of the birth weight among infants with same gender and gestational age. Birth outcomes data were extracted from medical records. Birth registrars who collect the data for the Perinatal Data System are instructed to first abstract medical records to obtain outcomes data, complemented with maternal interviews where necessary. Birth certificate data were used to derive the Adequacy of Prenatal Care Utilization (APNCU) Index that categorized women as follows: Adequate Plus (A+), Adequate, Intermediate, and Inadequate. The Index is based on the ratio of observed to expected (O/E) number of prenatal visits. The expected number of visits is based on the American College of Obstetricians and Gynecologists (ACOG) recommendations. In our analysis, we grouped Adequate Plus and Adequate as Adequate category and grouped intermediate and inadequate as the others category to create a binary variable for prenatal care. The grouping was completed to increase the power of the path analysis. Race and ethnic categories included in the PDS were as follows: White, Black, Asian, American Indian/Native Hawaiian, Other, Multiple races and Hispanic, Non-Hispanic ethnic categories. In our analyses, we used White vs. Non-White category, as a majority of the women in the PDS database were White, and to increase the power of the path analysis we made race a binary variable. Hispanic/Non-Hispanic category was not included in the final analyses, as it was not significantly associated with any of the outcome variables in the preliminary analyses.

### Statistical analysis

Characteristics of subjects with and without periodontal disease in the perinatal database were compared using Chi-Square tests for categorical variables and Student’s t-tests or Wilcoxon rank sum tests for continuous variables. Unadjusted effects of periodontal disease and depression on adverse outcomes such as small for gestational age (yes/no), large for gestational age (yes/no), preterm delivery (yes/no), and low birth weight (yes/no) were examined using both chi-square tests and logistic regression. Adjusted effects of periodontal disease and depression were estimated from multiple logistic regression models using SAS version 9.4 (SAS Institute, Inc., Cary, North Carolina) and path analysis using Mplus, version 7.3 (Muthen & Muthen, Los Angeles, California). The covariates adjusted in multiple logistic regression models and path analysis included mother’s age at delivery, parity, adequacy of health care during pregnancy (adequate plus, adequate, intermediate, inadequate), insurance type (Medicaid, Private insurance), mother’s race, tobacco use during pregnancy (yes/no), alcohol use during pregnancy (yes/no), and mother’s education level (less than high school, high school graduate or higher). A P-value <0.05 was considered statistically significant. Odds ratios (OR) and their 95% confidence intervals (CI) were calculated from both simple and multiple logistic regression models to assess the unadjusted and adjusted effects of periodontal disease and depression on adverse birth outcomes. The pathway analysis coefficients are the estimated coefficients from the logistic regression models in the pathway analysis that are equivalent to the log of estimated odds ratios.

## Results

### Characteristics of the study population

The distribution of maternal characteristics in the study population is presented in Table 1. The total number of women included in this analysis was 316,956. Almost 27% of women reported problems with their gums during pregnancy. A higher proportion of women who reported periodontal disease also reported that they experienced depressive symptoms during pregnancy, (40.09% versus 29.33% women without reporting periodontal problems, p-value<0.01). A higher proportion of women who reported periodontal disease, had adequate health care, were of white race, had private medical insurance, and reported alcohol use during pregnancy (p-values<0.01). A lower proportion of women who reported periodontal disease, reported tobacco use during pregnancy, were less educated, were younger at delivery and had lower parity (p-values< 0.01).

**Table 1:** Characteristics of subjects with/without periodontal disease in pregnancy data.

Variable	Periodontal Disease (*n* = 84238)	No Periodontal Disease (*n* = 232718)	*P* value*
**Depression (%)**			<0.01
** Yes**	33518 (40.09%)	67672 (29.33%)	
** No**	50098 (59.91%)	163050 (70.67%)	
**Adequacy of Care (%)**			<0.01
** Adequate Plus**	29238 (37.82%)	80593 (37.80%)	
** Adequate**	30052 (38.88%)	82099 (38.51%)	
** Intermediate**	9366 (12.12%)	23900 (11.21%)	
** Inadequate**	8644 (11.18%)	26606 (12.48%)	
**Mother's Race (%)**			<0.01
** White**	70741 (83.98%)	189413 (81.39%)	
** Non-White**	13497 (16.02%)	43305 (18.61%)	
**Type of Insurance (%)**			<0.01
** Medicaid**	31573 (40.71%)	89692 (41.66%)	
** Private Insurance**	45987 (59.29%)	125578 (58.34%)	
**Tobacco use (%)**			<0.01
** Yes**	10560 (12.54%)	37061 (15.93%)	
** No**	73652 (87.46%)	195601 (84.07%)	
**Alcohol use (%)**			<0.01
** Yes**	1022 (1.22%)	2418 (1.04%)	
** No**	83030 (98.78%)	229779 (98.96%)	
**Mother's Education (%)**			<0.01
** Less than high school**	10603 (12.60%)	36706 (15.80%)	
** High school graduate or higher**	73516 (87.40%)	195631 (84.20%)	
**Maternal Age at Delivery (mean, sd)**	27.38 (5.85)	27.71 (5.99)	<0.01
**Parity (mean, sd)**	1.43 (1.65)	1.64 (1.76)	<0.01

*P-values from Chi-square tests and t-test/Wilcoxon rank sum test are two-sided.

### Birth outcomes characterized by periodontal disease and depression

The distribution of birth outcomes characterized by maternal periodontal disease is presented in Table 2 and the distribution of birth outcomes characterized by maternal depression is presented in Table 3. Odds ratios (OR) and 95% confidence intervals (CI), based on the logistic regression model are also presented in Table 2 and Table 3. As presented in Table 2, women who reported periodontal disease any time during pregnancy more likely delivered babies that were large for gestational age than women who did not report periodontal disease (12.67% vs. 12.37%) with the OR = 1.029 and 95%CI (1.005–1.054). Women who reported periodontal disease any time during pregnancy less likely delivered babies that were small for gestational age (OR = 0.950, 95% CI 0.923–0.977), had preterm delivery (OR = 0.937, 95%CI 0.910–0.966), and less likely delivered babies that had low birth weight (OR = 0.933, 95%CI 0.902–0.966) when compared to women who did not report periodontal disease any time during pregnancy.

**Table 2:** Birth outcomes characterized by periodontal disease with unadjusted odds ratios and their 95% confidence intervals.

Variable	Periodontal Disease (*n* = 77247)	No Periodontal Disease (*n* = 211966)	*P* value*
**Small for gestational age**	OR = 0.950 (0.923, 0.977)	1	<0.01
**Yes**	6868 (8.16%)	19868 (8.55%)	
**No**	77285 (91.84%)	212543 (91.45%)	
**Large for gestational age**	OR = 1.029 (1.005, 1.054)	1	0.01
**Yes**	10663 (12.67%)	28744 (12.37%)	
**No**	73490 (87.33%)	203667 (87.63%)	
**Preterm delivery**	OR = 0.937 (0.910, 0.966)	1	<0.01
**Yes**	6231 (7.40%)	18292 (7.87%)	
**No**	77922 (92.60%)	214119 (92.13%)	
**Low birth weight**	OR = 0.933 (0.902, 0.966)	1	<0.01
**Yes**	4587 (5.45%)	13537 (5.82%)	
**No**	79651 (94.55%)	219180 (94.18%)	

*P-values from Chi-square tests are two-sided; OR estimates are from logistic regression models.

**Table 3:** Birth outcomes characterized by depression with unadjusted odds ratios and their 95% confidence intervals.

Variable	Depression (n = 94626)	No Depression (n = 195032)	*P* value*
**Small for gestational age**	OR = 1.347 (1.312, 1.382)	1	<0.01
**Yes**	10273 (10.06%)	16500 (7.68%)	
**No**	91842 (89.94%)	198237 (92.32%)	
**Large for gestational age**	OR = 0.872 (0.852, 0.892)	1	<0.01
**Yes**	11675 (11.43%)	27745 (12.92%)	
**No**	90440 (88.57%)	186992 (87.08%)	
**Preterm delivery**	OR = 1.231 (1.197, 1.265)	1	<0.01
**Yes**	8968 (8.78%)	15573 (7.25%)	
**No**	93147 (91.22%)	199164 (92.75%)	
**Low birth weight**	OR = 1.390 (1.348, 1.434)	1	<0.01
**Yes**	7142 (6.98%)	10994 (5.11%)	
**No**	95135 (93.02%)	203974 (94.89%)	

*P-values from Chi-square tests are two-sided; OR estimates are from logistic regression models.

As presented in Table 3, women who reported depressive symptoms during pregnancy more likely delivered babies that were small for gestational age (OR = 1.347, 95%CI 1.312–1.382), had preterm delivery (OR = 1.231, 95% CI 1.197–1.265) and more likely delivered babies that had low birth weight (OR = 1.390, 95%CI 1.348–1.434) compared to women who did not report depressive symptoms during pregnancy.

### Path analysis

**Fig 1:**
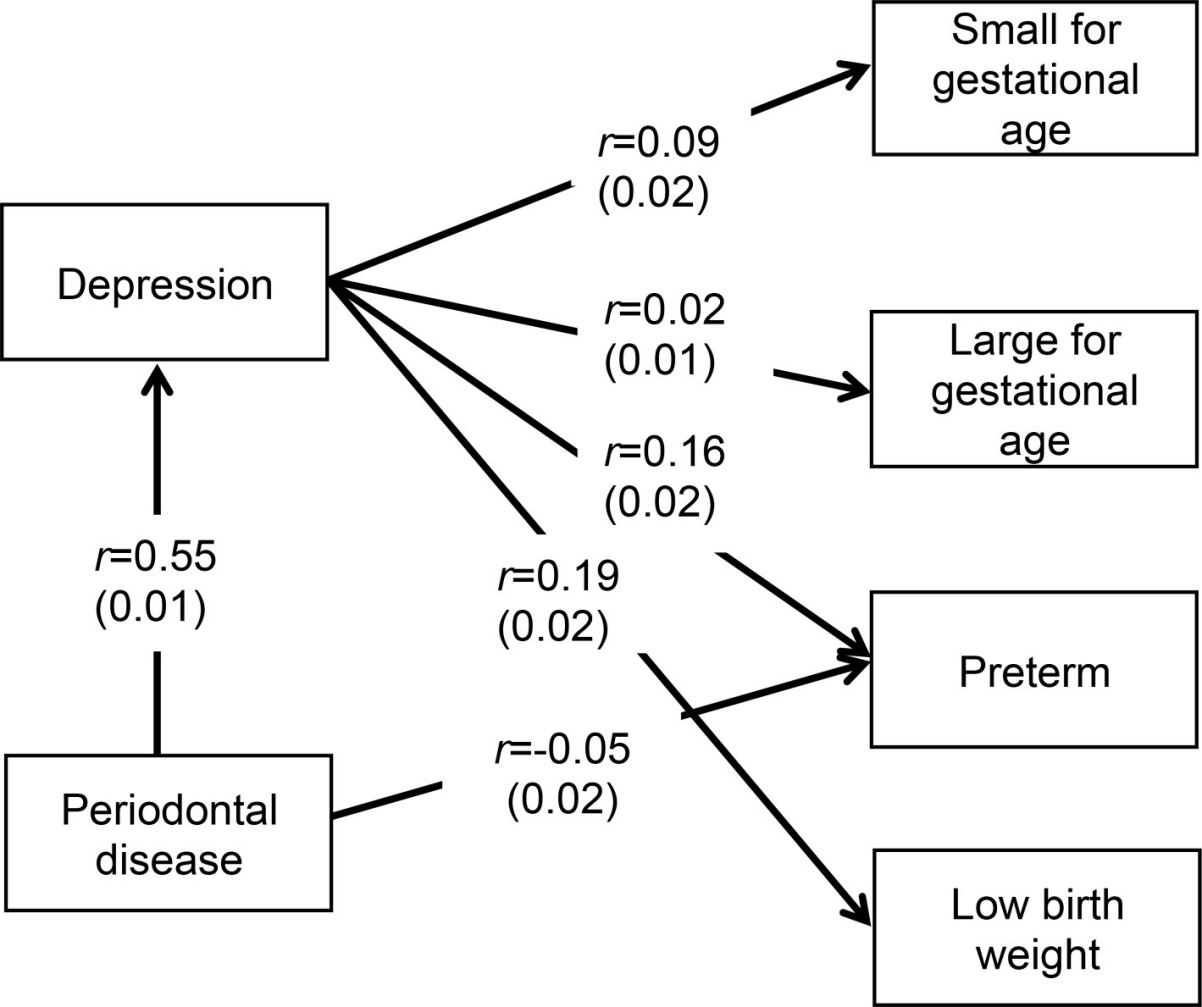
Significant Coefficients and standard deviations for periodontal disease, depression, and adverse birth outcomes from path analysis after adjusting for mother’s age, parity, adequate health care during pregnancy, insurance type, mother’s race, tobacco use during pregnancy, alcohol use during pregnancy, and mother’s education level.

The results from path analysis are presented in Table 4 and Fig 1. The total number of women included in this analysis was 269,990. The associations of adverse birth outcomes with periodontal disease, depression and mother’s characteristics were evaluated via the logistic regression portion of the path analysis. Estimated ORs and 95% CIs are presented in Table 4. Several adverse birth outcomes such as being small for gestational age, being large for gestational age, having preterm delivery and having low birth weight were associated with depression, periodontal disease and mother’s characteristics. For example, being small for gestational age was significantly associated with depression (OR = 1.093, 95% CI 1.066–1.121), smoking (OR = 2.469, 95% CI 2.402–2.537) and alcohol use during pregnancy (OR = 1.190, 95% CI 1.085–1.304), and being less educated (OR = 1.247, 95% CI 1.210–1.284). Being large for gestational age was significantly associated with having adequate health care (OR = 1.034, 1.011–1.058), private insurance (OR = 1.053, 95% CI 1.029–1.078), being white (OR = 1.870, 95% CI 1.814–1.927), and being older (OR = 1.022, 95% CI 1.020–1.024), and having higher parity (OR = 1.086, 95%CI 1.080–1.092). Having preterm delivery was associated significantly with depression (OR = 1.177, 95%CI 1.146–1.208), having adequate health care (OR = 1.638, 95% CI 1.589–1.689), smoking during pregnancy (OR = 1.259, 95% CI 1.220–1.300), being less educated (OR = 1.214, 95% CI 1.174–1.256), being older (OR = 1.003, 95% CI 1.001–1.006), and having higher parity (OR = 1.037, 95% CI 1.029–1.044). Having low birth weight was significantly associated with depression (OR = 1.206, 95% CI 1.170–1.243), having adequate health care (OR = 1.397, 95% CI 1.351–1.445), smoking during pregnancy (OR = 1.855, 95% CI 1.793–1.919), using alcohol during pregnancy (OR = 1.214, 95% CI 1.086–1.356), and being less educated (OR = 1.322, 95% CI 1.275–1.370). Depression was significantly associated with periodontal disease (OR = 1.728, 95% CI 1.702–1.756), smoking during pregnancy (OR = 1.762, 95% CI 1.727–1.797), using alcohol during pregnancy (OR = 1.470, 95% CI 1.377–1.570), being less educated (OR = 1.125, 95% CI 1.102–1.149), and having higher parity (OR = 1.065, 95% CI 1.060–1.069). Periodontal disease was significantly associated with being white (OR = 1.192, 95% CI 1.167–1.217) and using alcohol during pregnancy (OR = 1.314, 95% CI 1.227–1.407).

**Table 4:** Estimated odds ratios and their 95% confidence intervals from path analysis (n = 269990).

Variable	Small for gestational age	Large for gestational age	Preterm delivery	Low birth weight	Depression	Periodontal Disease
	OR (95% Confidence Interval)	OR (95% Confidence Interval)	OR (95% Confidence Interval)	OR (95% Confidence Interval)	OR (95% Confidence Interval)	OR (95% Confidence Interval)
**Depression**						
** Yes**	1.093 (1.066, 1.121)	1.017 (0.995, 1.039)	1.177 (1.146, 1.208)	1.206 (1.170, 1.243)		
** No**	1	1	1	1		
**Periodontal Disease**						
** Yes**	0.981 (0.955, 1.008)	0.998 (0.976, 1.020)	0.950 (0.923, 0.977)	0.964 (0.932, 0.996)	1.728 (1.702, 1.756)	
** No**	1	1	1	1	1	
**Adequate Care**						
** Yes**	0.970 (0.946, 0.995)	1.034 (1.011, 1.058)	1.638 (1.589, 1.689)	1.397 (1.351, 1.445)	0.956 (0.940, 0.972)	0.977 (0.960, 0.994)
** No**	1	1	1	1	1	1
**Primary Payer**						
** Private Insurance**	0.788 (0.767, 0.810)	1.053 (1.029, 1.078)	0.906 (0.880, 0.932)	0.808 (0.782, 0.835)	0.658 (0.647, 0.669)	0.927 (0.910, 0.943)
** Medicaid**	1	1	1	1	1	1
**Race**						
** White**	0.533 (0.519, 0.548)	1.870 (1.814, 1.927)	0.820(0.796, 0.846)	0.598 (0.579, 0.618)	0.802 (0.787, 0.817)	1.192 (1.167, 1.217)
** Non-White**	1	1	1	1	1	1
**Smoke during pregnancy**						
** Yes**	2.469 (2.402, 2.537)	0.410 (0.395, 0.425)	1.259 (1.220, 1.300)	1.855 (1.793, 1.919)	1.762 (1.727, 1.797)	0.771 (0.753, 0.788)
** No**	1	1	1	1	1	1
**Alcohol use during pregnancy**						
** Yes**	1.190 (1.085, 1.304)	0.792 (0.713, 0.880)	1.075 (0.966, 1.195)	1.214 (1.086, 1.356)	1.470 (1.377, 1.570)	1.314 (1.227, 1.407)
** No**	1	1	1	1	1	1
**Education**						
** Less than high school**	1.247 (1.210, 1.284)	0.816 (0.788, 0.844)	1.214 (1.174, 1.256)	1.322 (1.275, 1.370)	1.125 (1.102, 1.149)	0.771 (0.753, 0.790)
** High school graduate or higher**	1	1	1	1	1	1
**Mother' age in years**	0.995 (0.993, 0.998)	1.022 (1.020, 1.024)	1.003 (1.001, 1.006)	1.002 (0.999, 1.005)	0.970 (0.968, 0.971)	0.989 (0.988, 0.991)
**Parity**	0.912 (0.905, 0.919)	1.086 (1.080, 1.092)	1.037 (1.029, 1.044)	0.987 (0.979, 0.996)	1.065 (1.060, 1.069)	0.953 (0.948, 0.958)

In Fig 1, we present results from path analysis as coefficients and standard deviations after adjusting for mother’s age, parity, adequate health care during pregnancy, insurance type, mother’s race, tobacco use during pregnancy, alcohol use during pregnancy and mother’s education level. Periodontal disease was significantly associated with depression (r = .55) and preterm delivery (r = .05). Depression was significantly associated with being small for gestational age (r = 0.09), being large for gestational age (r = 0.02), having preterm delivery (r = 0.16) and low birth weight (r = 0.19) in the population under study. We concluded that a positive association existed between depression during pregnancy and adverse birth outcomes, and that depression served as a mediator in the association of periodontal disease with adverse birth outcomes.

## Discussion

Our findings obtained from a large perinatal database in the Finger Lakes region of NY State suggest that depression during pregnancy is associated with all adverse birth outcomes under study, including being small for gestational age, being large for gestational age, being preterm and having low birth weight. Depression often remains untreated during pregnancy despite the fact that the prevalence of psychosocial stress is substantial [11]. Contrary to earlier views, pregnancy is not protective against a major depressive episode that may be dangerous to the pregnant woman and the baby. The vast burden of depression on women, their children and their families has been well-acknowledged over the past twenty years [20], however there is a need for serious, rigorously conducted research into effective and safe treatment for depression in women, particularly at times over the course of the of reproductive years. A systematic review reported that prevalence rates for depression assessed by the validated screening instruments were 7.4%, 12.8% and 12.0% for the first, second, and third trimesters, respectively [21]. The authors of the review concluded that rates of depression, especially during the second and third trimesters of pregnancy were substantial and that clinical and economic studies to estimate maternal and fetal consequences were essential. In the current study, 32.7% of women reported depression during pregnancy (Table 2). Women, who reported depressive symptoms during pregnancy more likely delivered babies that were small for gestational age, had preterm delivery and delivered babies that had low birth weight when compared to women who did not report depressive symptoms during pregnancy. Our findings are in agreement with a systematic review and meta-analysis on depression and adverse birth outcomes [10] and also with the results of a large community based birth cohort study and a prospective cohort study conducted in Holland and the US [11, 12]. A recent literature review that assessed the risk of adverse pregnancy outcomes and perinatal and neonatal complications of the offspring related to in utero exposure to antidepressants suggested antidepressant exposure was associated with fetal growth changes and shorter gestations, although effects were small [22].

Positive associations between periodontal disease and adverse birth outcomes are well documented in the literature, however treatment of periodontal disease during pregnancy does not prevent preterm birth, fetal growth restriction, or preeclampsia [2–5, 23]. In our study, periodontal disease was indirectly associated with adverse birth outcomes via depression but women with periodontal disease were more likely to be depressed. Self-reported periodontal disease in our study was not associated directly with adverse birth outcomes (Table 4). Our study results related to periodontal disease and adverse pregnancy outcomes are in agreement with the results of the multicenter prospective cohort study conducted in three hospitals in Philadelphia PA [8]. The authors of the aforementioned study concluded that they did not observe an association between the presence of periodontal disease and composite adverse pregnancy outcomes, including preeclampsia, preterm birth, intrauterine growth restriction and perinatal death. Davenport and colleagues reported that after adjustment for maternal age, ethnicity, maternal education, smoking, alcohol consumption, infections, and hypertension during pregnancy, they found no evidence for an association between preterm low birth weight and periodontal disease in a case-control study [7]. There are potential explanations for inconsistencies in the literature related to the association between periodontal disease and adverse pregnancy outcomes, including varying criteria used to define periodontal disease, timing of the assessment and, as in our study, self-reported data that may underestimate or overestimate disease prevalence and which cannot differentiate between transient (pregnancy related) periodontal bleeding and inflammation, and adult periodontitis. Furthermore, race and education level may contribute to differential prevalence rates of periodontal disease, as periodontal disease is more prevalent among men, Mexican Americans, adults with less than a high school education, adults below 100% Federal Poverty Levels and current smokers [24]. A majority of women in this study were white and had a higher educational attainment. By definition, this group had a lower prevalence of periodontal disease, which might further attenuate the association between periodontal disease and adverse birth outcomes. Moreover, it is not known whether women who self-reported periodontal disease during pregnancy had sought dental treatment. Recent reanalysis of the Obstetrics and Periodontal Therapy (OPT) trial data concluded that periodontal treatment among mothers with mild to moderate periodontal disease before 21 weeks of gestation may prevent preterm births [25]. Women of childbearing age should be examined by dental practitioners regularly and screened for periodontal disease and if the disease is present, they should be offered treatment options appropriate to the level of disease observed.

Our results also suggest that adverse birth outcomes were associated with smoking and alcohol use during pregnancy. Smoking and alcohol use during pregnancy are detrimental for the developing fetus and pregnancy outcomes; our analyses suggest that they were both harmful for pregnancy outcomes; women who reported smoking during pregnancy and use of alcohol were more likely to have preterm delivery and deliver babies with low birth weight. Reporting socially undesirable lifestyles occurring during pregnancy may have resulted in under-reported, yet significant findings. The key strength of our study was the analysis of data from a large, well-characterized sample from the general population of women who delivered babies in the Finger Lakes region of New York State, thereby enhancing the robustness and generalizability of the findings. A further strength was the availability of birth outcome data, including detailed information on infant weight, gestational age and preterm or term delivery of the baby. Limitations, in addition to the cross-sectional nature of the study, included the self-reported information about periodontal status and depression during pregnancy, and lack of data on use of anti-depressive medications. Thus, data must be interpreted cautiously with respect to the potential for recall bias and over- or under-estimation of the findings. Although the PDS database is broadly representative, our data may not be generalizable beyond the Finger Lakes region of New York State.

We conclude that a positive association exists between depression during pregnancy and adverse birth outcomes, and that depression serves as a mediator in the association of periodontal disease with adverse birth outcomes. Considering the theorized link between infection, inflammation and adverse pregnancy outcomes, it is biologically plausible to consider depression as a mediator in the association of periodontal disease with adverse pregnancy outcomes. Potential pathways identified may include behavioral, infectious, endocrine and inflammatory mechanisms [26], especially given emerging evidence suggesting that inflammatory responses have an important role in the pathophysiology of depression [14] and that periodontal disease is one of the most prevalent chronic infectious diseases that lead to a chronic systemic inflammatory response [8].

Preterm and low birth weight infants are at greater risk for mortality and a variety of health and developmental problems. As a result, the birth of a preterm or low birth weight infant may have significant emotional and economic effects on the infant’s family. The infant mortality rate for low birth weight infants is about 25 times that of the infant mortality rate for normal weight babies [27]. Rates of low birth weight and preterm birth may differ by maternal race/ethnicity. Black women have consistently had higher rates of preterm and low birth weight babies [28].

Considering the results of our study, depression may serve as a mediator in the association of periodontal disease with adverse birth outcomes. As such, obstetricians and dental providers caring for pregnant women should be made aware that women who have periodontal issues are more likely to be depressed during pregnancy and more likely to experience adverse birth outcomes. Future studies should include prospective studies that focus on oral health assessment of expectant mothers, administration of diagnostic interviews to assess depression during pregnancy, consideration for the use of anti-depressive medications during pregnancy, and counseling related to detrimental health behaviors such as smoking and alcohol use during pregnancy.
